# Supplementary material for: Backscattering amplitude in ultrasound localization microscopy
Source: Sci Rep. 2023 Jul 16;13:11477. doi: 10.1038/s41598-023-38531-w (PMC10350458; doi:10.1038/s41598-023-38531-w)
Supplement: Supplementary file 1 — Supplementary Figure 1. [file 41598_2023_38531_MOESM1_ESM.pdf]

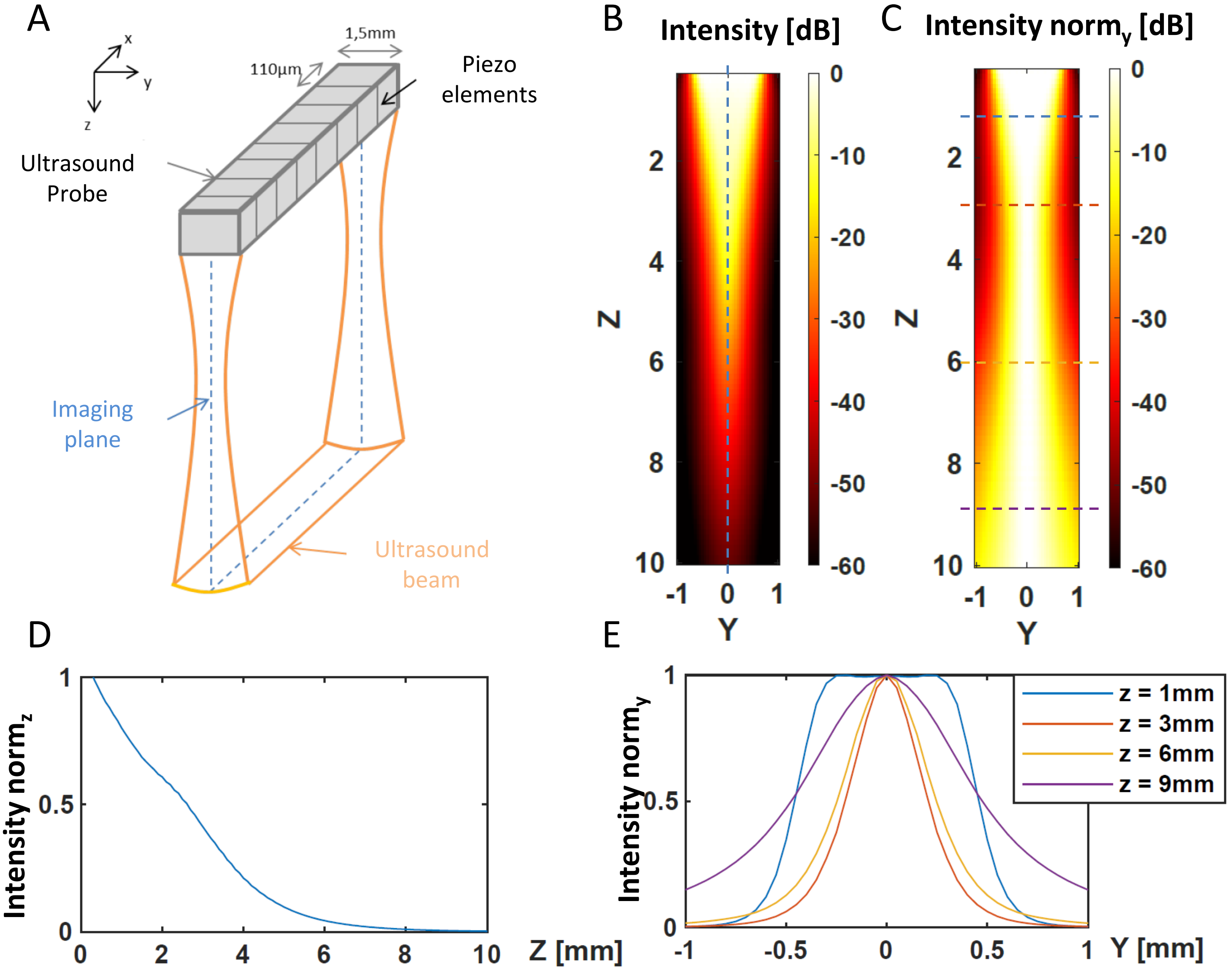

Figure suppl 1 : Simulation of the ultrasound beam pressure field and Intensity. (A) Schematic of the ultrasound probe and characteristic spatial dimensions. **(B)** Ultrasound Intensity in the YZ plane at the X central position (X = 0). **(C)** Ultrasound Intensity in the YZ plane (X = 0) normalized for each depth z. **(D)** Decrease of ultrasound intensity with depth z (**I**(x=0, y=0, z) along the blue dashed line in B). **(E)** Ultrasound intensity profile along y for a given depth, normalized for each depth as in C (along colored lines in C).
